# Spatially Broadband Coupled-Surface Plasmon Wave Assisted Transmission Effect in Azo-Dye-Doped Liquid Crystal Cell

**DOI:** 10.3390/nano10071357

**Published:** 2020-07-11

**Authors:** Guan-Ting Dong, Chun-Ta Wang, Yu-Ju Hung

**Affiliations:** Department of Photonics, National Sun Yat-sen University, Kaohsiung 804, Taiwan; qq308025@gmail.com (G.-T.D.); ctwang@mail.nsysu.edu.tw (C.-T.W.)

**Keywords:** surface plasmon polariton, polymer dispersed liquid crystal, Mie scattering, extraordinary transmission, grating

## Abstract

Active tuning on a plasmonic structure is discussed in this report. We examined the transient transmission effects of an azo-dye-doped liquid crystal cell on a metallic surface grating. The transition between isotropic and nematic phases in liquid crystal generated micro-domains was shown to induce the dynamic scattering of light from a He-Ne laser, thereby allowing transmission through a non-transparent aluminum film overlaying a dielectric grating. Various grating pitches were tested in terms of transmission effects. The patterned gratings include stripe ones and circular forms. Our results indicate that surface plasmon polariton waves are involved in the transmission process. We also demonstrated how momentum diagrams of gratings and Surface Plasmon Polariton (SPP) modes combined with Mie scattering effects could explain the broadband coupling phenomenon. This noteworthy transition process could be applied to the development of spatially broadband surface plasmon polariton coupling devices.

## 1. Introduction

Since the optical transmission effects produced by an array of holes in metallic materials was observed [1,2,3], different optical branches were developed quickly [4]. A variety of imaginative designs based on “flat land” optics have tapped into the power of bounded two-dimensional surface plasmon polaritons (SPPs) [5,6,7,8]. Well-defined hyperbolic multi-film structures and advances in material design (e.g., meta-materials and transformation optics) have made it possible to customize the optical dielectric constant [9,10,11]. In the tuning perspective, the optical performance of soft liquid-crystal materials or other optical tunable materials on nano-metallic structures has been widely reported [1,12,13,14,15,16,17,18,19,20]. Soft liquid-crystal materials are easily manipulated and highly effective in the active tuning of optical parameters. The superior optical properties of SPPs (i.e., strong surface E-field, short interaction length in the coupling region, short effective wavelength, and compact mode profile) can be exploited by actively tuning the upper dielectric environment. The large δn difference of LC molecules makes them highly sensitive to the effects of polarization, which means that they could be potentially used in the fabrication of polarization-sensitive metamaterials [21,22]. Many reports have demonstrated the SPPs can be tuned by altering the liquid crystal (LC) layer [12,17,18,23,24]. Previous reports have computed complex SPP modes and demonstrated SPP diffraction effects [14,25]. A switching voltage of ~5 V is sufficient to affect these devices. However, the pinning of LC molecules to the surface tends to degrade tuning contrast. In this study, we sought to prevent these pinning effects by sandwiching a photosensitive liquid crystal layer between an ITO glass superstrate and an undulating Al film. Al plasmonics were studied and shown to be cheap, able to provide broadband resonance, and feasible in color filter designs [12,18,26,27,28,29]. Here, the periodic gratings beneath the film are used as coupling components in the generation of SPPs. The photosensitive LC layer can be switched between isotropic and nematic phases. During this transition, the micro-boundaries created between isotropic and nematic phases scatter the incident light, which subsequently undergoes coupling to form surface plasmons. Due to the scattering effect which possesses broad bandwidths both in frequency domain and spatial frequency domain, aluminum plasmonic structure is more suitable than the conventional gold and silver structure in which the plasmonic resonance bandwidth shows more limited in spectrum. Detailed analysis of the scattering process of photo-sensitive LC materials can be found in [30,31]. Despite extensive research into the light scattering effects of dielectric materials and metallic particles, the near field of phase-transition micro boundaries is a crucial issue that must be elucidated formally. A similar sample structure has been reported in [17], where the polymer-dispersed liquid crystal droplets were patterned as fixed gratings filled between the two substrates. The refractive index of the fixed gratings could be changed by photo-tuning techniques. The optical performance in [17] is a mixed phenomenon between the thick soft material gratings and the patterned Au gratings. The interaction between localized surface plasmon polariton wave (LSPP) and the fixed LC gratings is attributed to the reported switching effect. In this report, we focus primarily on the interaction between the Mie scattering effect from the LC phase transition boundaries and long range SPP modes [32] which results in the large transient transmission effect.

The photosensitive material used in this study was an azobenzene nematic liquid crystal (azo-NLC) comprising two isomers, namely a rod-like trans-isomer and a bent cis-isomer [18,33,34,35,36]. Note that the thermodynamic stability of trans-isomers typically exceeds that of cis-isomers. Photo-induced isomerization (involving exposure to ultraviolet or violet light) can be used to induce trans- to cis- isomerization. The reverse isomerization involving spontaneous thermal relaxation can be accelerated by exposure to light at visible (i.e., longer) wavelengths. The trans- to cis- isomerization of azo molecules disturbs the order of the azo-NLC, thereby lowering the nematic–isotropic phase transition temperature, which depends on the concentration of the cis-isomer associated with the azo molecules. This means that when the operating temperature is set between the clearing points of azo-LC (in a cis-rich-state or trans-rich-state), the azo-NLC can be photo-isothermally switched between nematic and isotropic phases. Phase switching results in the formation of micro-boundaries, which can induce Mie scattering. This in turn expands the range of in-plane k-vectors, which greatly enhances the likelihood that light will be coupled into SPPs and subsequently re-transmitted through the metallic structure. We postulate that it should be possible to use these effects to create a broadband SPP coupling device operating in the spatial frequency domain. The reported phenomenon here has also pointed out the possible sensing applications as in [37]. In the Discussion section, momentum k-vector diagrams are used to illustrate the observed transmission effects.

## 2. Materials and Methods

### 2.1. Sample Preparation

**Figure 1:**
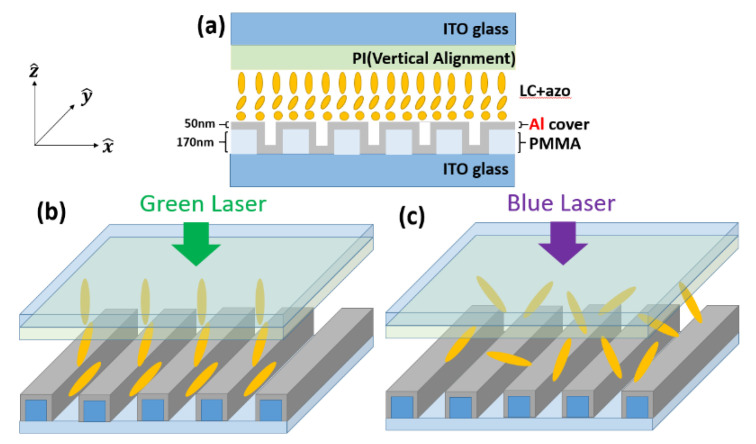
(**a**) Sample structure: LC aligned in HAN mode; (**b**) Green pumping laser switching azo dye to final trans-state in which the LC molecules are aligned in hybrid mode; (**c**) Blue laser switching azo dye molecules to final cis-state, wherein the LC layer is isotropic.

**Figure 2:**
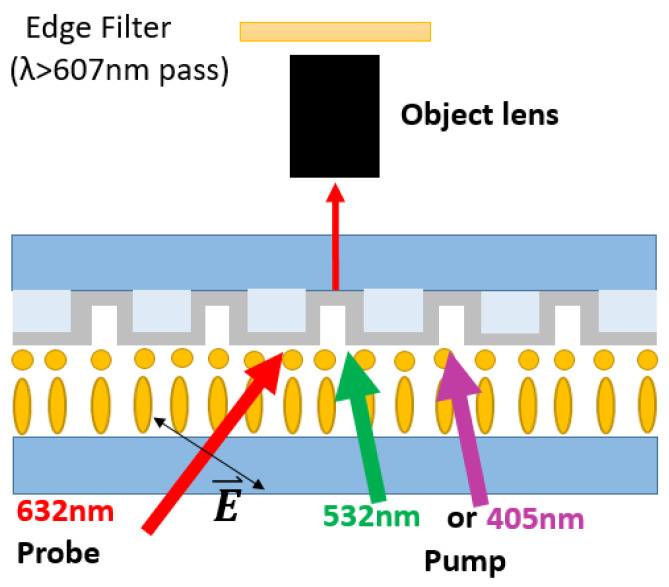
Setup of optical microscope. The pump and probe light were incident to the side of the cell and the signal transmission through the Al was collected by the microscope. The LC alignment is in HAN mode.

Figure 1 presents a schematic illustration showing the structure of the samples examined in this paper. Two ITO glass substrates (upper and lower) were used to sandwich a single liquid crystal layer (5CB) above a poly(methyl methacrylate) (PMMA) grating covered by a film of Al (50 nm). Note that the LC was in hybrid alignment nematic (HAN) mode. The LC molecules proximal to the upper rubbing layer were aligned vertically, whereas the LC molecules close to the grating were aligned horizontally (i.e., with the grating trench). Photo-tuning in the direction of the LC molecules was achieved by mixing azo-NLC (1205, Beam Co., Anniston, AL, USA) into the LC host at a ratio of 20/80 wt%. Trans- and cis- isomerization of the azo dye molecules was used to induce a phase transition in the LC host between nematic and isotropic phases. As shown in Figure 2, the phase transitions were respectively triggered using green and blue lasers. When the sample was irradiated by blue light, the azo-LC was switched from trans-rich-state to cis-rich-state, and the nematic–isotropic transition temperature drops from 43.9 °C to 13.0 °C. The LC operating at room temperature thus switched to isotropic phase. Note that this process was faster than the reverse process (i.e., inducing the nematic phase via green light illumination). In addition, the isotropic phase can stably exist for hours upon the removal of blue light irradiation, owing to the slow spontaneous cis–trans relaxation of 1205 [38]. Based on our previous experience [14], as shown in Figure 6, the effective working pitches for LC material on Au gratings are around 300~400 nm. The k_spp_ value at 632.8 nm for Al/LC interface (structure in this report) is similar to that at Au/LC interface @ 533 nm. Therefore, we chose the grating pitches around 300~400 nm.

### 2.2. Measurements

Figure 2 illustrates the setup of the optical microscope. All light was incidental to the side of the cell, and the signal transmitted through the Al film was collected by an optical microscope (OM) with 10× objective (NA 0.3). The probe light (HeNe Laser @632.8 nm/1 mW) was TM polarized at an incident angle of 45 degrees. A longpass edge filter was used to filter out the pumping light (532 nm/5 mW and 405 nm/3 mW) to produce a clear background.

## 3. Results and Discussion

**Figure 3:**
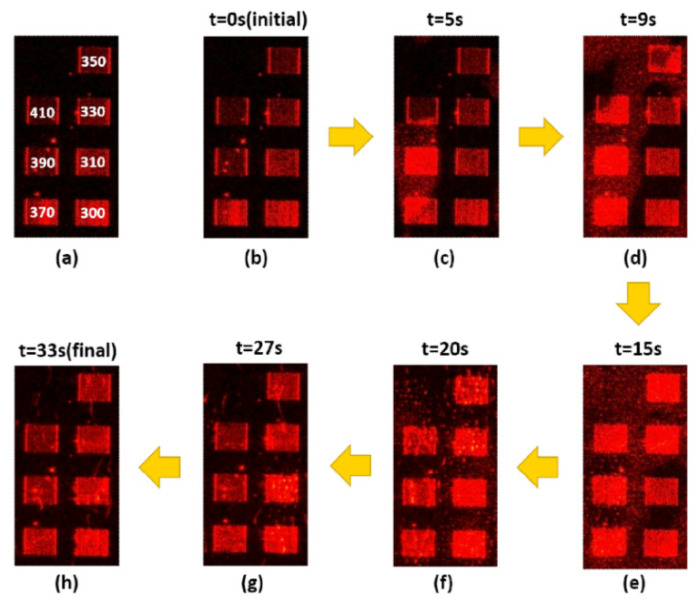
Sequential images from (**a**–**h**) illustrating transitional transmission through Al grating samples with pitches ranging from 300 nm to 410 nm. The pumping light is 532 nm. Liquid crystals are transformed from isotropic phase to nematic phase.

**Figure 4:**
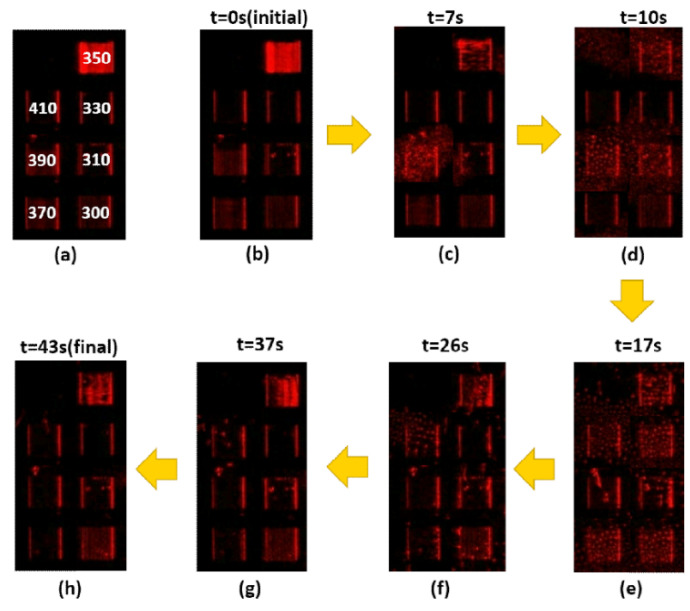
Images obtained from control samples with Ni gratings instead of Al grating (but with the same periodicity), showing no signs of transmission from (**a**–**h**). The pumping light is 532 nm. Liquid crystals are transformed from isotropic phase to nematic phase.

**Figure 5:**
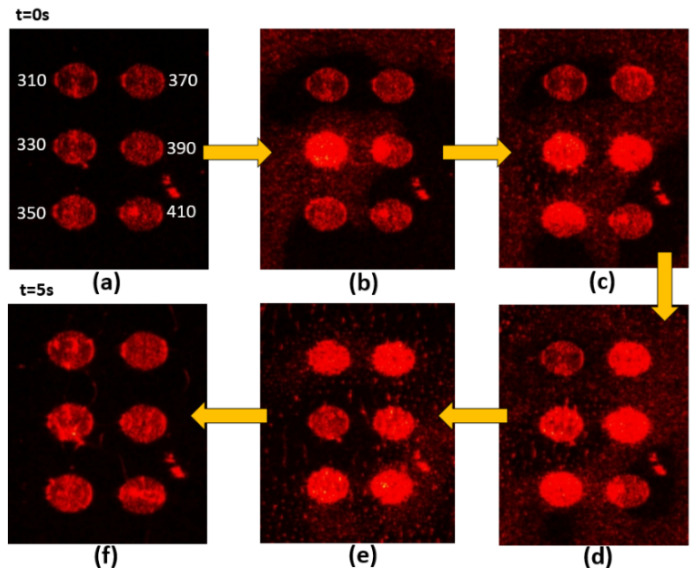
Sequential images from (**a**–**f**) illustrating transitional transmission through Al circular grating samples with pitches ranging from 310 nm to 410 nm. The pumping light is 532 nm. Liquid crystals are transformed from isotropic phase to nematic phase.

**Figure 6:**
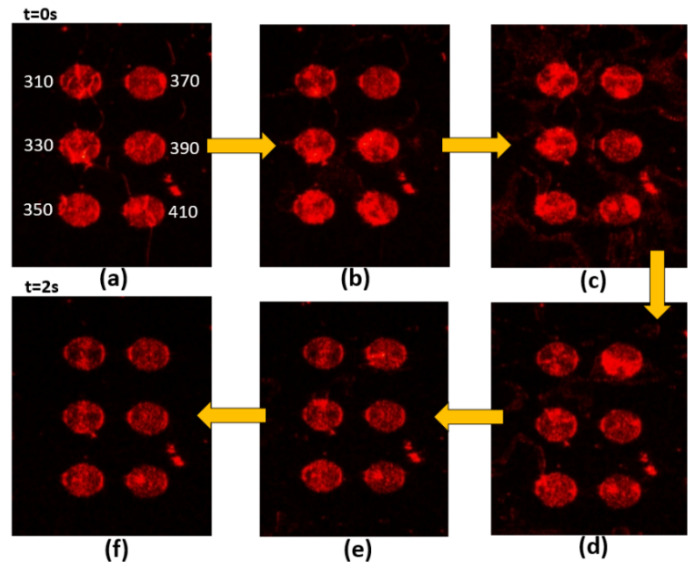
Sequential images from (**a**–**f**) illustrating transitional transmission through Al circular grating samples with pitches ranging from 310 nm to 410 nm. The pumping light is 405 nm. Liquid crystals are transformed from nematic phase to isotropic phase.

Figure 3, Figure 4, Figure 5 and Figure 6 present the transmission effects that occurred during phase transition process. As shown in Figure 3a, we selected stripe-grating pitches ranging from 300 to 410 nm, based on previous analysis indicating that these values would satisfy the quasi-phase matching conditions required to excite surface plasmon waves on an Al film [14]. The cell gap of the LC layer on stripe gratings and circular ones are around 8 and 6 microns respectively. The k-vectors of Mie scattered light covered a wide range of angles, and the reciprocal vectors of the grating were added in. These effects induced scattering across a wide range of directions to facilitate transmission. This scattering mechanism loosened the strict SPP coupling requirements, such that light with one specific incident angle could be used to induce SPP coupling in all of the gratings (regardless of pitch). Internal reflection at micro-boundaries is another important mechanism involved in SPP coupling. The fact that all of the evanescent waves (regardless of direction) are adjacent to the Al gratings makes them ideal for SPP coupling. Note that complex near-field issues are beyond the scope of this paper. Figure 4 outlines a control experiment in which the Al layer was replaced by a layer of Ni. Under these conditions (with the Ni film), we did not observe SPP resonance (or transmission effects) under excitation using visible light (i.e., 532 nm). The cis-state to trans-state was induced by illuminating 532 nm on the sample as reported in Figure 3, Figure 4 and Figure 5 due to slow process while the reverse transition happened quickly in 2 s regime as shown in Figure 6. The transmission of some of the gratings decreased after *t* = 15 s in Figure 3. Defect annihilation and the relaxation of domain boundaries could be the reason for this trend.

**Figure 7:**
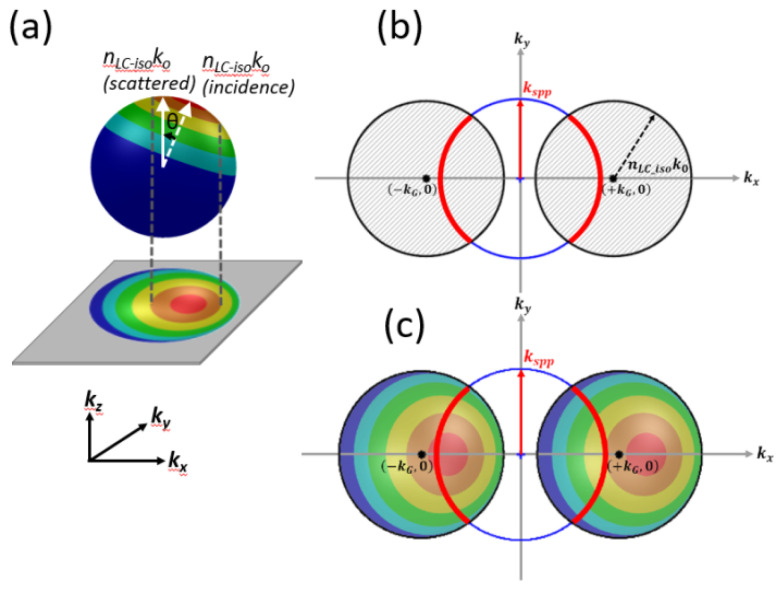
(**a**) k-sphere showing Mie scattering in photo-sensitive LC cell layer. The scattering angle can be derived from the colored projection map; (**b**) Mie scattering (full-circle) modulated by reciprocal grating vector (+1 and −1 order). The red arcs represent the k vectors of Mie scattering fitted to k_spp_ vectors; (**c**) Mie scattering (area map) showing regions of strong and weak coupling under Mie scattering.

The scattering of light by particles exceeding the wavelength of the light can be attributed to Mie scattering. Scattering efficiency depends on the size of the particle, the refractive index contrast between the particle and its environment, the incident wavelength, and the propagation directions of the scattered light. Please refer to [39] for details pertaining to scattering efficiency. Figure 7a presents the vectors of waves scattered by a particle (denoted as a Mie scattering event). The k vector of the incident light in LC was denoted as n_LC-iso_*k_o_ (incidence). The angle between n_LC-iso_*k_o_ (incidence) and light in the scattered direction was defined as θ, and k_z_ was parallel to the optical column of the OM during observation. Generally, forward scattering (i.e., colorful regions other than the blue hemisphere) tends to be more pronounced, whereas backward scattering (i.e., the blue region) decays to nearly zero when θ is increased to 180 degrees. The zones with a smaller solid angle w.r.t. the axis of n_LC-iso_*k_o_ (incidence) are associated with higher scattering efficiency.

The in-plane components of scattered light on the k_x_–k_y_ plane are related directly to SPP excitation. The lower part of Figure 7a presents a projection of the colored zones onto the k_x_–k_y_ plane. The colored sections indicate the degree of Mie scattering efficiency w.r.t. scattered solid angle θ. It is clear that scattering events occurred in every direction, such that the projection of the colored area appears as a “filled” k circle indicating the possible existence of all in-plane k vectors.

Figure 7b shows how the scattered in-plane k vectors were modulated by the planar Al gratings, with the result that the filled circle with radius n_LC-iso_*k_o_ was shifted by +/− k_G_ (=2π/pitch). The red arcs in Figure 7b represent all possible k-vectors associated with SPP waves excited by scattering components, as modulated by the Al grating. k_spp_ was computed from the PMMA (*n* = 1.5)/Al planar interface system. The exact value of k_spp_ are listed in Table 1. Figure 7c is an imposition of the color map in Figure 7a, showing the efficiency of Mie scattering events. Arcs passing through regions of higher scattering efficiency (e.g., red/orange regions) were prone to k_spp_ excitation, whereas the rims in areas of lower scattering efficiency (e.g., blue regions) were less prone to SPP excitation. The k_spp_ arcs located near the red/orange regions indicate that more of the light was coupled into SPP waves, resulting in the transmission of more of the light. In Figure 7c, k_G_ shifting indicates higher transmission. Differences in grating pitch manifest as differences in shift length (i.e., the absolute value of k_G_) in the colored circles. Thus, the grating pitch could be specified to ensure that the k_spp_ circle intersects with the filled colored circles. Table 1 shows the computed k_spp_ values on Al and Ni films based on different data bases [40,41,42,43,44]. The average value of k_spp_ at 633 nm is around 1.6*k_o_. The imaginary part of k_spp_ on Ni film is 6 times more loss than that on aluminum film. For the initial state shown in Figure 4b, the Ni grating with 350 nm pitch shows high transmission. Most incidence light plus grating k_G_ vector could be coupled to the anti-symmetrical mode described in [14]. Meanwhile, during the scattering process, the scattered light coupled into SPP mode for Ni film is very insufficient and lossy.

**Figure 8:**
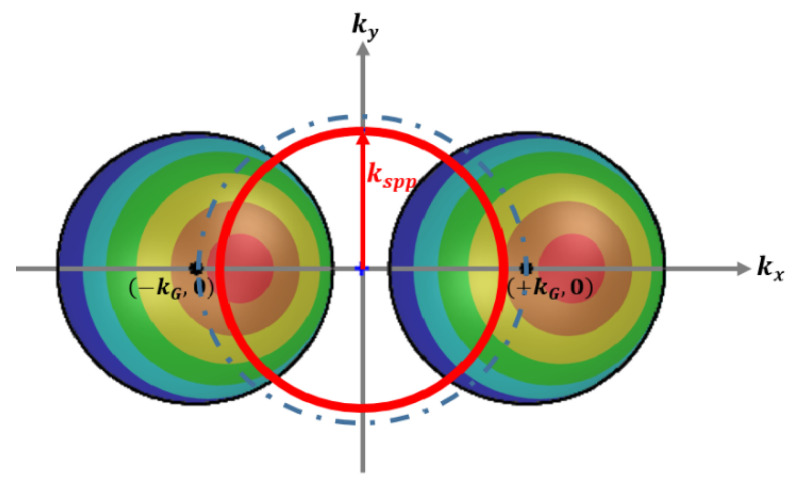
The Mie scattering k vector modulated by the reciprocal k_G_ vector of a circular grating. The colorful Mie scattering vector span is moving around the dotted circular outline with radius k_G_. The red circular k_spp_ boundary could intersect with the colorful Mie scatting area while the color area is moving around the dotted line.

Figure 8 represents the scattering event modulated by circular gratings. The Mie scattering-filled circle area could move around the dotted circular outline that represents the k_G_ vector of the circular grating. In the meantime, the red highlighted k_spp_ circle could intersect with the Mie scattering color area, which represents the Mie scattering k vectors modulated by the circular grating k_G_ vector could meet the excitation criterion for surface plasmon polariton. It is worth noting that k_G_ vector length could be in a wide range due to different grating pitches, while the color circle area scanned around the dotted line (with radius k_G_) could excite the plasmonic modes. The interaction time much shorter on circular gratings than the stripe ones shown in Figure 3, Figure 5, and Figure 6 might be the following: Firstly, the cell gaps of stripe gratings and circular ones are 8 and 6 microns, respectively. The thickness of LC layer might affect the transition time slot. Secondly, there are higher chances for the scattering components matching k_spp_ by adding up k_G_ with 360° span from circular gratings. Thirdly, the original alignment of stripe grating substrate and circular one are different. The circular one somehow favors the boundary turning on process. The short interaction time on circular gratings actually indicates that this scheme is more suitable for sensor applications since it is more effective in time and more sensible for the refractive index change on the surface.

## 4. Conclusions

This paper described the transient transmission effects of an azo-dye-doped liquid crystal layer on a subwavelength metallic grating. The transition of the LC layer between isotropic and nematic phases produced numerous boundaries, which resulted in Mie scattering. Coupling imposed by short period gratings enabled the transmission of scattered light through the Al-coated gratings, regardless of the grating pitch. This loosened the momentum matching conditions associated with surface plasmon wave assisted transmission effects. These findings indicate the possibility of creating broadband spatial frequency coupling devices, as long as the transient micro-boundaries could be fixed in the time domain.

## Figures and Tables

**Table 1:** Computed k_spp_ values on Al film and Ni film from various data bases. The database values could be found in [45].

**Aluminum**	**Database (at λ = 632 nm)**	**Real(k_spp_)/k_o_**	**Imag(k_spp_)/k_o_**
Data1	Rakić (1995)	1.625	0.014
Data2	Rakić: Brendel-Bormann model (1998)	1.628	0.013
Data3	McPeak (2015)	1.635	0.016
**Nickel**	**Database (at λ = 632 nm)**	**Real(k_spp_)/k_o_**	**Imag(k_spp_)/k_o_**
Data1	Johnson and Christy (1974)	1.649	0.085
Data2	Rakić: Brendel-Bormann model (1998)	1.647	0.108
Data3	Werner: DFT calculations (2009)	1.632	0.071
